# Validation of the Arabic Version of Calgary Depression Scale for Schizophrenia

**DOI:** 10.1371/journal.pone.0162304

**Published:** 2016-09-01

**Authors:** Yahya Hani, Suhaila Ghuloum, Ziyad Mahfoud, Mark Opler, Anzalee Khan, Arij Yehya, Abdulmoneim Abdulhakam, Samer Hammoudeh, Azza Al-Mujalli, Reem Elsherbiny, Hassen Al-Amin

**Affiliations:** 1 Department of Psychiatry, Rumailah Hospital, Hamad Medical Corporation, Doha, Qatar; 2 Department of Health Policy and Research, Weill Cornell Medicine - Qatar, Doha, Qatar; 3 Prophase, LLC, New York, United States of America; 4 Department of Research, Weill Cornell Medicine - Qatar, Doha, Qatar; 5 Primary Health Care Corporation, Doha, Qatar; 6 Department of Psychiatry, Weill Cornell Medicine - Qatar, Doha, Qatar; SPAIN

## Abstract

**Background:**

Patients with schizophrenia commonly show both depressive and negative symptoms that can differentially affect the prognosis and course of treatment. The Calgary Depression Scale for Schizophrenia (CDSS) was designed to distinguish between depression and negative symptoms in patients with schizophrenia. The purpose of this study is to validate an Arabic version of the CDSS among patients with schizophrenia.

**Methods:**

The diagnosis of schizophrenia was confirmed using the Arabic Mini International Neuropsychiatric Interview 6 (MINI 6). A standardized translation back-translation process was adopted. One rater administered the Arabic CDSS to subjects with schizophrenia as well as to a control group who should not have any psychiatric disorder except for depression. Another rater, blinded to the results administered the already validated Arabic version of Beck Depression Inventory-II (BDI-II).

**Results:**

We recruited 102 patients and 102 controls subjects. The CDSS showed good internal consistency in the active group (Cronbach’s alpha = 0.82). The Intraclass Coefficient correlations (ICC) for the inter-rater reliability (n = 21) was 0.90, *p*<0.05 and test-retest reliability (n = 19) was 0.85, *p*<0.001. When compared to the BDI-II, the cutoff score of 5 on the Arabic CDSS showed reasonable sensitivity and specificity of 72.75% and 67.95% respectively.

**Conclusions:**

The psychometric properties of the Arabic version of CDSS demonstrate that it is a valid tool to assess the depressive symptoms in the Arab patients with schizophrenia.

## Introduction

Schizophrenia is a widespread chronic mental disorder affecting 26.3 million worldwide as reported by World Health Organization (WHO) [1]. In addition to the positive and negative symptoms of the disorder, depressive mood is significantly higher in patients with schizophrenia compared to healthy population [2].

Diagnosing depression in patients with schizophrenia is still a challenge since it is difficult to differentiate between depressive and negative symptoms in schizophrenia [3, 4]. Some features might help to distinguish between these two overlapping entities e.g. blunted affect is a negative symptom whereas hopelessness, guilt, and suicidal ideation are mostly depressive symptoms. However, other symptoms may be more difficult to distinguish such as anhedonia, social withdrawal and lack of energy [4].

Many scales have been used to assess depression in schizophrenia but these were originally validated in patients with depression not schizophrenia. In 1990 a group of researchers developed a scale to measure depression among patients with schizophrenia [5]. The scale was derived from items in the Hamilton Depression Rating Scale (HAM-D) and the Present State Examination (PSE) [5]. This 9-item scale is now known as the Calgary Depression Scale for Schizophrenia (CDSS). The validity and internal consistency of the CDSS are known to be high [6]. Furthermore, the CDSS shows a low correlation with the negative symptoms subscale of the Positive and Negative Syndrome Scale (PANSS), which supports its ability to differentiate depression from negative symptoms [7]. In a systematic review [8] comparing the different scales used to assess depression in schizophrenia [CDSS, HAM-D, Beck Depression Inventory (BDI-II) and depression items of the PANSS], the authors concluded that CDSS demonstrated the best psychometric properties.

CDSS is now available in 38 different languages and has been validated in several cultures including Greek [9], French [10], Spanish [11], German [12], Chinese [13], Brazilian [14] and Thai [15], where significant validity and reliability measures was demonstrated in all cases.

To date, there are no studies that have assessed depressive symptoms in Arab patients with schizophrenia.

The goals of this study were to validate the Arabic version of the CDSS, measure its psychometric properties and report preliminary data on depressive symptoms in the Arab patients with schizophrenia residing in Qatar.

## Methods

This study is part of a larger project on the validation of Arabic versions of several scales, including the PANSS, which are used in the assessment and treatment of patients with schizophrenia. This paper reports results in relation to the validation of the Arabic version of the CDSS.

### Subjects

The subjects were recruited from the Arab population residing in Doha, Qatar. This country is witnessing, in the last few years, a rapid economic development that is associated with a substantial growth in the population. The Arabs constitute about 26.75% of the total population in Qatar; of those, 45% are Qatari and 55% are non-Qatari Arabs [16]. The study team recruited 109 subjects with schizophrenia with or without depression, but 7 cases were dropped because they did not complete all the required scales. The controls were 102 healthy individuals, with no psychiatric diagnosis. The selected sample size is adequate for the estimation of sensitivity and specificity to within a margin of error of at most 9% at a 95% confidence interval. It is also consistent with the number of patients used in the previous studies that validated the translated scale in other languages such as French, German, and Spanish [10–12]. The patients were recruited from the inpatient wards at the Department of Psychiatry, Rumailah hospital in Doha, Qatar. The controls were enrolled through the primary healthcare centers in Doha and from relatives and friends of the patients with schizophrenia. All subjects signed an Informed Consent Form (ICF). The Joint Institutional Review Board at Hamad Medical Corporation (HMC) and Weill Cornell Medicine-Qatar (WCM-Q) approved the study. The study was conducted between January 2013 and January 2015.

All the subjects first underwent a semi-structured interview using the Arabic version of the Mini International Neuropsychiatric Interview 6 for schizophrenia and other psychotic disorders (MINI 6) [17] to confirm the presence or absence of psychiatric DSM-IV-TR diagnosis including schizophrenia and/or depression.

The inclusion criteria for active cases were: DSM-IV-TR diagnosis of schizophrenia with or without depression, male or female between 18 and 65 years of age, having Arabic as their native language and they or their family being able to read, understand and sign the ICF. The exclusion criteria were: history of current or past (over the last six months) drug or alcohol abuse, presence of DSM-IV-TR diagnosis other than schizophrenia, hearing or vision impairment, slurred or incomprehensible speech and at high risk of harming themselves or others. The inclusion and exclusion criteria for the controls were the same as above except that subjects did not have diagnosis of schizophrenia or other psychiatric disorders.

### Translation Procedure

A committee of three bilingual psychiatrists and one professional translator independently conducted the translation from English to Arabic. They then deliberated and agreed on the first version. This version was piloted in a sample of 20 subjects (10 with schizophrenia and 10 who were healthy controls) to check if this Arabic version was clear and understandable to subjects and interviewers. The committee assessed the feedback from the pilot group, made further changes and agreed on the semi-final Arabic version. Another independent professional translator back translated the latter scale into English. The committee reviewed the back translation together with the author of the original scale. Further changes were made to the Arabic version as per the feedback from the author until we had his final approval on the back translation.

### Study design

One rater was responsible for screening the subjects' eligibility as well as confirming the schizophrenia diagnosis using MINI 6 for schizophrenia and other psychotic disorders. The same rater also obtained the socio-demographic information and the medical and psychiatric history of the patient. In addition to that, this rater administered the validated Arabic version of BDI-II [18, 19]. A second rater, who was blinded to the results and information obtained by the first rater, administered the Arabic versions of CDSS and PANSS. In 21 cases, a third rater attended the interview with the second rater and independently scored the CDSS and PANSS to assess the inter-rater reliability. For test-retest reliability, 19 patients were again given the CDSS and PANSS after 3 days by the same rater. Raters who administered the scales had extensive training and supervision before the start of the research project. For the CDSS, they completed at least five practice sessions before the administration of the scale with the research subjects.

### Statistical analysis

Demographic and other clinical characteristics were summarized using means and standard deviations for continuous variables such as age, and frequency distributions for categorical data such as gender and educational level. Comparisons between the groups were done using t-test for continuous measures and chi-square for categorical ones. Internal consistency of the Arabic CDSS was assessed on 102 cases using Cronbach’s alpha. As per Nunnally, the recommendation is for alpha to be above 0.70, where the values 0.7–0.8 are considered moderate and 0.8–0.9 are high [20]. The intraclass correlation coefficient (ICC), in 2-way random manner with absolute agreement type, was used to measure the inter-rater reliability (n = 21 cases) and test-retest reliability (n = 19 cases). ICC values between 0.40 and 0.59 are considered fair, values between 0.60 and 0.74 are good and between 0.75 and 1.0 are excellent [21]. According to BDI-II instructions, the scores can be classified into low, moderate or high to specify the degree of depression. To determine the best sensitivity and specificity cutoff points of the Arabic CDSS to determine the presence of depression in patients with schizophrenia, the Receiver Operating Characteristics (ROC) curve was used to compare the CDSS composite scores with those having moderate to high values on BDI-II. Principal Component Analysis (PCA) was carried out on the CDSS scores of the patients with schizophrenia to identify subsets of symptoms and their contributions to the variance. Construct validity was tested by the correlation between scores on the CDSS and the BDI-II. The correlation between CDSS total score and the negative subscale of the validated Arabic PANSS [22] was done to test the divergent validity. The convergent validity was tested by measuring the Pearson’s correlation between CDSS score and the depression item in the Arabic PANSS (G6). All analyses were done using the Statistical Package for Social Science (IBM-SPSS, version 23). Statistical significance was set at the level of 0.05.

## Results

### Sample Characteristics

The schizophrenic patients had an average age of 35.2 (SD = 9.99) years, (range 18–62 years), with the majority being males (66.7%), Qatari (61.8%) and single (58.8%). These latter three frequencies were significantly higher in the patients (*p*<0.05) compared to the control group. No significant age differences were found between the two groups (Table 1).

**Table 1:** Demographic Characteristics.

	Patients (*n* = 102)	Controls (*n* = 102)
**Age (Mean(SD))**	35.17 (9.99)	33.97 (8.26)
**Gender**		
**Male**	68 (66.7%)^a^	43 (42.2%)
**Female**	34 (33.3%)	59 (57.8%) ^b^
**Country Born**		
**Qatari**	63 (61.8%) ^a^	35 (34.3%)
**Non-Qatari**	39 (38.2%)	67 (65.7%) ^b^
**Marital Status**		
**Married**	28 (27.5%)	78 (76.5%) ^b^
**Single**	60 (58.8%) ^a^	22 (21.6%)
**Divorced**	12 (11.8%)	2 (2.0%)
**Widowed**	1 (1.0%)	-
**Missing**	1 (1.0%)	-
**Education Level**		
**Never Attended School**	2 (2.0%)	-
**Intermediate or Elementary School**	37 (36.3%)	9 (8.8%)
**Secondary or High School**	40 (39.2%) ^a^	16 (15.7%)
**Vocational Degree**	2 (2.0%)	15 (14.7%)
**College Degree or Post- Graduate University Degree**	21 (20.6%)	62 (60.8%) ^b^
**Employment**		
**Employed**	30 (29.5%)	101 (99%) ^b^
**Student**	9 (8.8%)	1 (1%)
**Unemployed**	63 (61.7%) ^a^	-

^a^ Significantly more in schizophrenia group (*p*<0.05).

^b^ Significantly more in the control group (*p*<0.05).

More subjects in the control group had achieved college or post-graduate degree (60.8%) and were employed (99%) when compared to the schizophrenia group (*p*<*0*.*05*).

The median number of inpatient admissions in the patient group was 3 and ranged from 0 to 18. The mean age at the onset of psychotic symptoms was 23.06 (SD = 8.30) years. The mean age of first admission was 26.33 (SD = 7.77) years. The mean duration of illness was 9.40 (SD = 9.17) years. In the schizophrenia group 37.3% of patients reported a history of aggressive behavior and 30.4% gave a history of suicide attempts.

Using the BDI cutoff for moderate/ severe depression, the patient group had significantly higher percentage of subjects with depression (21.6%) than the control group (3.9%). Among the patients with schizophrenia, none were receiving benzodiazepines, 70 (68.6%) were on antipsychotics and only 6 (5.9%) were on antidepressants. None of the subjects in the control group were receiving antipsychotics or benzodiazepines and only one was taking an antidepressant.

### Reliability Measures

The Internal consistency of the Arabic CDSS in the patient group (n = 102) was considered high as the Cronbach’s alpha is 0.82 [20] (Table 2). The CDSS total score ranged from 0 to 22 with an average of 4.17 (SD = 4.3). The inter-rater reliability and test-retest reliability were reasonably high with (ICC = 0.90, p<0.05 and ICC = 0.85, p<0.001) respectively. Both ICC values suggest excellent agreement as per Cicchetti [21].

**Table 2:** Calgary Depression Scale for Patients with Schizophrenia (*n* = 102).

	Mean	S.D.	Cronbach alpha if item was deleted
**1. Depressed mood**	0.69	0.96	0.79
**2. Hopelessness**	0.44	0.71	0.81
**3. Self-Depreciation**	0.42	0.71	0.79
**4. Guilty ideas of Reference**	0.49	0.70	0.79
**5. Pathological guilt**	0.41	0.67	0.80
**6. Morning Depression**	0.41	0.72	0.78
**7. Early wakening**	0.37	0.76	0.81
**8. Suicide**	0.36	0.74	0.79
**9. Observed Depression**	0.62	0.76	0.79
**Total**	4.19	4.3	0.82

ICC was done on active group. Also, ICC was done in a 2-way random manner with absolute agreement type, and then average measure was taken.

### Validity Measures

**Fig 1:**
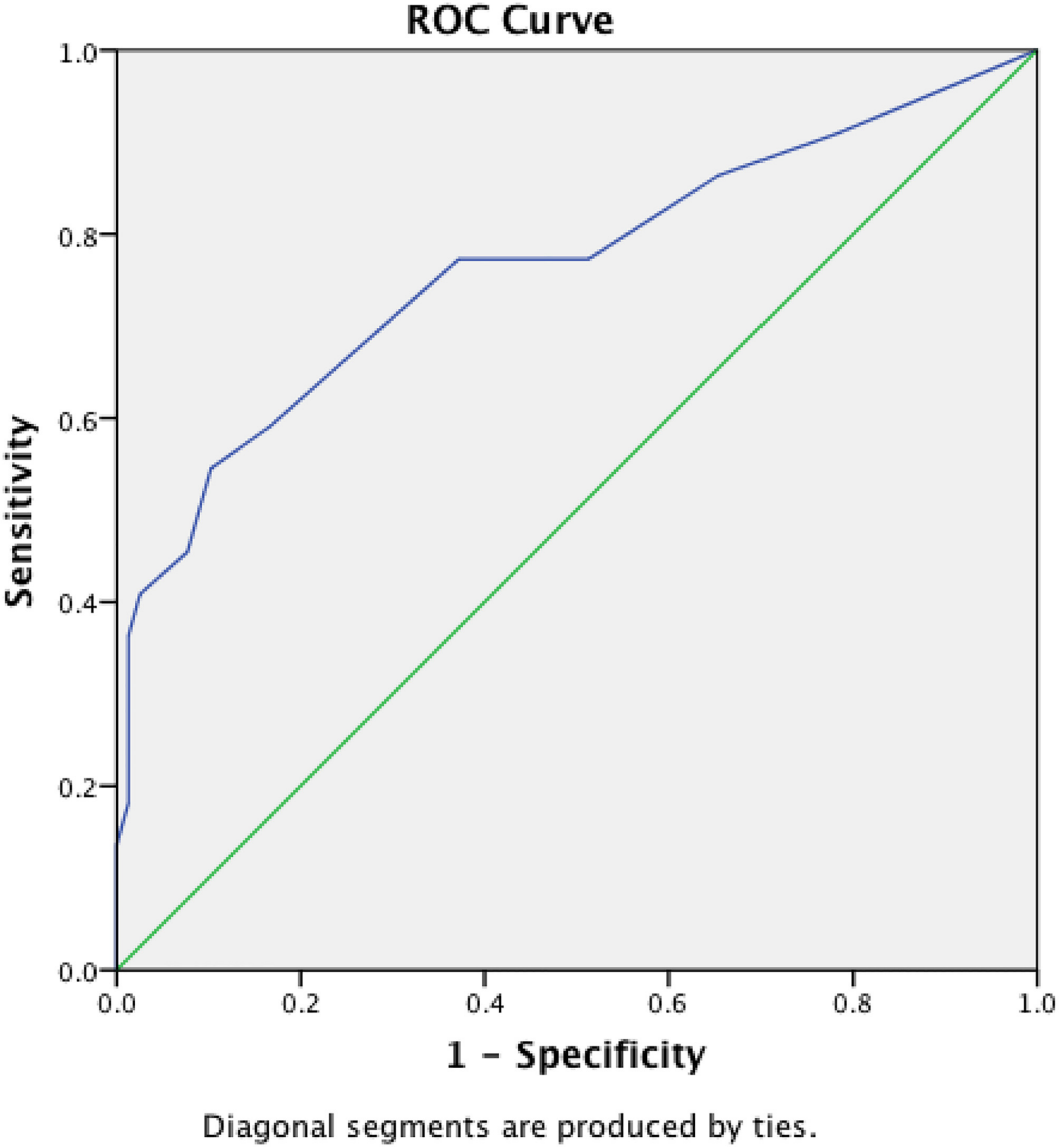
ROC Curve. The Arabic CDSS total score with the Arabic BDI-II cutoff score for moderate/ severe depression as gold standard.

On the ROC curve (Fig 1), the best cutoff value was 5 with 72.75 sensitivity and 67.95 specificity. The area under the ROC curve was 0.764 (95% CI 0.635–9.894, *p*<0.001). Using the cutoff of 5 on the CDSS, to distinguish between depressed vs. non-depressed subjects, the prevalence of depression in the schizophrenia group was 36.3% (37 subjects) while only 8.8% (9 subjects) of the control group would qualify for a diagnosis of depression.

The construct validity was significantly good as measured by the correlation between the total scores on Arabic CDSS and BDI-II (r = 0.63, *p*<0.001). Convergent validity was also acceptable as the correlation between the PANSS-G6 (the item for depression) and CDSS total score was high (r = 0.36, *p*<0.001). According to Cohen [23], the effect size for the Pearson correlation was good for the construct validity and medium for the convergent one.

We tested divergent validity by the correlation between CDSS and PANSS negative subscale (r = 0.17) and it was not significant suggesting that CDSS items do not overlap with the PANSS measures of negative symptoms.

### Principal Component Analysis

PCA revealed two factors (Table 3) that explained 54.44% of variance. The PCA had eigenvalue over Kaiser criterion of 1 [24] and the variance explained by factors 1 and 2 were 41.15% and 13.29%, respectively. Factor 1 included the following items: depressed mood, guilty ideas of reference, pathological guilt, morning depression, early awakening and observed depression. Factor 2 included the items on hopelessness, self-depreciation and suicide (Table 3).

**Table 3:** Principal Component Analysis.

	Component	
	Factor 1	Factor 2
**1. Depressed mood**	0.66	
**4. Guilty ideas of Reference**	0.64	
**5. Pathological guilt**	0.48	
**6. Morning Depression**	0.72	
**7. Early wakening**	0.78	
**9. Observed Depression**	0.59	
**2. Hopelessness**		0.84
**3. Self-Depreciation**		0.77
**8. Suicide**		0.55
**Total Variance Explained**	41.15%	13.29%

Total Variance Explained: 54.44%

KMO = 0.76, Bartlett’s Test of Sphericity *p*<0.001.

## Discussion and Limitations

### Discussion

The results of this study demonstrate that the Arabic version of the CDSS has good psychometric properties as assessed by different reliability and validity measures. The mean score for Arabic patients with schizophrenia of 4.19 (SD = 4.3) is very close to the one reported in the original CDSS [6]. Other versions of CDSS like the French, Greek or Thai have reported a mean score that ranged from 3.88 (SD = 4.05) to 6.97 (SD = 4.82) [9, 10, 15, 25]. The prevalence of depression (36.3%) in our schizophrenia group using a cutoff of 5 on CDSS was higher than that in the control group (8.8%) or that reported in the Qatari population (13.3%) [26]. This is in accordance with other studies [27, 28].

The internal consistency was acceptable, (Cronbach’s alpha = 0.82) which is in line with the high consistency of other validation studies as in Chinese (0.80) [13], Thai (0.869) [15], Greek (0.87) [9], Spanish (0.83) [11], Brazilian (0.80) [14], French (0.82) [10] and Japanese (0.82) [29]. The range of Cronbach’s alpha for the other versions of CDSS is 0.76–0.88 (see review by Lako et al. [30]). Our results showed that all items in the scale are essential, which has also been demonstrated in the Thai [15] and Spanish [11] versions. However other studies showed that item 3 (self-depreciation) in the Greek version [9], items 4 (guilty ideas of reference) and 7 (early morning awakening) in the French one [10] and items 4, 6 (morning depression) and 7 in the Chinese version [13] were not necessary as they showed weak correlation with the other items or with the total CDSS score. This variability in the different cross-cultural validation studies could be related to sampling of patients, severity of their illness, specific manifestations of depression across cultures and the type of medications they are receiving.

The Arabic version of CDSS has clear instructions, does not require much clinical training and can be quickly mastered by raters as shown by the high inter-rater reliability (ICC = 0.90), which is distinctly higher than those reported in the Chinese (0.79) [13], Greek (0.78) [9] or Spanish (0.73) [11] studies. The range of agreement reported by Lako et al. for other studies of CDSS is 0.73–0.98 [30]. The test-retest reliability was high and within the range reported for the original and other versions of CDSS [30].

The criterion validity of the Arabic CDSS was assessed by measuring the sensitivity and specificity for depression at different CDSS cutoff points and using the score on Arabic BDI-II as a reference. The cutoff score that gave the best values for sensitivity and specificity was 5. This score is in agreement with other cross cultural studies that used the DSM clinical diagnosis of depression like the Spanish [11] and Greek [9] versions. However, the Thai [15] French [10] and Brazilian [14] versions gave the best criterion validity at the score 6/7; and still others [8] reported that the optimal cutoff score was 8/9. The different cutoff scores could be related to the cultural differences in the depressive symptoms in patients with schizophrenia or due to using different methods to make the diagnosis of depression.

The positive correlation between CDSS and PANSS-G6 or total score of BDI demonstrates the convergent validity of the Arabic CDSS. The original CDSS and many of the other CDSS versions have demonstrated positive correlations with other scales for depression like the BDI [6, 8] the HAM-D [9] and others (see review by Lako et al. [30]).

Divergent validity for the Arabic version of CDSS was shown by the weak and insignificant correlation between the Arabic CDSS and the negative subscale of the PANSS in patients with schizophrenia, thus confirming that the Arabic CDSS can distinguish the depressive symptoms from the negative ones of schizophrenia. Although the author of the original CDSS [6] reported a significant correlation between CDSS and negative PANSS subscale, many subsequent validation studies reported no correlation with this subscale [8–10, 13, 14].

PCA of the Arabic CDSS items revealed two factors that accounted for 54.44% of the total variance. Factor 1 explained 41.15% of the variance and included the items covering the major features of depression; items # 1, 4, 5, 6, 7 and 9. Factor 2 explained 13.29% of total variance and covered the items # 2, 3 and 8 that could be interpreted as all relating to suicidal ideation. The French CDSS also showed two factors with very similar variances as ours but the second factor included two different items (guilty ideas of reference and early wakening) [10]. However, two studies [31, 32] on stable outpatients with chronic schizophrenia reported three factors with total variance greater than 65% where factor 2 included guilty ideas of reference and pathological guilt while factor 3 included only early wakening. Some of these differences in factor loadings could be explained by the type of samples studied (acutely ill vs. stable patients) and the cultural differences in the presentation of symptoms of depression in patients with schizophrenia. For example, in the Arab culture, talking about loss of hope and thoughts of suicide is considered taboo because of religious sensitivities.

### Limitations

The results should be interpreted with caution because of few limitations. First, the patient group included more males and thus there could be a gender issue. Schizophrenia is known to have worse prognosis in males than females [33] who usually need less number of admissions [34]. The active group was mostly inpatients and this might explain why the majority were males in our sample. Another possible explanation is that the population in Qatar is predominantly male (75.6%) [16]. Second, more subjects in the control group achieved higher levels of education. Other studies have shown that higher educational level might be protective against depression [35]. A related concern here is that Arabs, who have different dialects, often do not routinely use the formal Arabic. Third, the majority in subjects examined were Qataris and as such our findings may not be generalizable to all Arab countries. Thus, more studies in the different Arab countries may be needed to confirm the psychometric properties of the Arabic CDSS.

## Conclusion

Depression is more common in Arab patients with schizophrenia compared to healthy population. The Arabic version of CDSS is a valid and reliable tool to assess depression in this population in clinical settings and for research purposes. It can also adequately differentiate the depressive symptoms from the negative ones and thus can better guide the clinicians treating these symptoms in patients with schizophrenia.
